# Charge-Modulated Synthesis of Highly Stable Iron Oxide Nanoparticles for In Vitro and In Vivo Toxicity Evaluation

**DOI:** 10.3390/nano11113068

**Published:** 2021-11-14

**Authors:** Sunyoung Woo, Soojin Kim, Hyunhong Kim, Young Woo Cheon, Seokjoo Yoon, Jung-Hwa Oh, Jongnam Park

**Affiliations:** 1 School of Energy and Chemical Engineering, Ulsan National Institute of Science and Technology (UNIST), Ulsan 44919, Korea; sywoo1225@unist.ac.kr (S.W.); khh2008@unist.ac.kr (H.K.); 2 Department of Predictive Toxicology, Korea Institute of Toxicology (KIT), Daejeon 34114, Korea; sjkim@kitox.re.kr; 3 Department of Bio and Brain Engineering, Korea Advanced Institute of Science and Technology (KAIST), Daejeon 34141, Korea; 4 Department of Plastic and Reconstructive Surgery, Gachon University Gil Medical Center, Incheon 21565, Korea; youngwooc@gilhospital.com; 5 Department of Human and Environmental Toxicology, University of Science and Technology (UST), Daejeon 34113, Korea; 6 Departmento of Biomedical Engineering, Ulsan National Institute of Science and Technology (UNIST), Ulsan 44919, Korea

**Keywords:** iron oxide nanoparticles, toxicity, biocompatibility, colloidal stability PEG ligands, PEG ligands

## Abstract

The surface charge of iron oxide nanoparticles (IONPs) plays a critical role in the interactions between nanoparticles and biological components, which significantly affects their toxicity in vitro and in vivo. In this study, we synthesized three differently charged IONPs (negative, neutral, and positive) based on catechol-derived dopamine, polyethylene glycol, carboxylic acid, and amine groups, via reversible addition–fragmentation chain transfer-mediated polymerization (RAFT polymerization) and ligand exchange. The zeta potentials of the negative, neutral, and positive IONPs were −39, −0.6, and +32 mV, respectively, and all three IONPs showed long-term colloidal stability for three months in an aqueous solution without agglomeration. The cytotoxicity of the IONPs was studied by analyzing cell viability and morphological alteration in three human cell lines, A549, Huh-7, and SH-SY5Y. Neither IONP caused significant cellular damage in any of the three cell lines. Furthermore, the IONPs showed no acute toxicity in BALB/c mice, in hematological and histological analyses. These results indicate that our charged IONPs, having high colloidal stability and biocompatibility, are viable for bio-applications.

## 1. Introduction

Iron oxide nanoparticles (IONPs) can be useful in applications such as magnetic resonance imaging contrast agents and biosensors and in various biomedical fields, such as hyperthermia treatment, drug delivery, cancer targeting, transfection, cell tracking, and tissue repair, owing to their low toxicity, unique magnetic properties, and facile surface modification [1,2,3,4,5,6]. To use IONPs for bio-applications, the IONPs should have excellent colloidal stability, functionalization with biomolecules, and non-toxicity under physiological conditions [7].

Through surface modification, IONPs can be fabricated to be stable and biocompatible in biological environments and have useful functional groups capable of binding to various biomolecules [8,9,10]. However, during the surface modification process, the surface physicochemical properties of IONPs, such as the hydrodynamic diameter (HD), shape, porosity, and surface charge, are inevitably changed. Among these properties, changes in the surface charge of IONPs significantly affect the cytotoxicity because they directly affect the interaction between the charged IONPs and biological components [11].

Several research groups have studied the effects of charged IONPs on cytotoxicity, genotoxicity, and neurotoxicity [12,13,14,15,16]. However, charged IONPs suffer from limitations related to their colloidal stability and the chemical structure of their surface ligands. For example, charged IONPs can induce nonspecific interactions with proteins, thus promoting other interactions between themselves and cells and leading to protein corona [17,18]. The protein corona increases the HD of the nanoparticles, thus leading to their precipitation. Furthermore, particle uptake, pharmacokinetics, and bio-distribution lead to increased toxicity of the nanoparticles [19,20]. In addition, the toxicity evaluations in studies on charged IONPs vary because of the different chemical structures of the ligand. Rivet et al. [21] demonstrated that charged IONPs fabricated using aminosilane, dextran, and poly-(dimethylamine-co-epichlorohydrin-co-ethylenediamine) exhibit cortical neuron cytotoxicity and showed a correlation between the metabolic activity and surface charge. However, the properties of the nanoparticles, except for the surface charge induced by the varieties of amine, alcohol, and zwitterion ligands on the surfaces, can have more unpredictable effects on cytotoxicity [22,23,24]. Therefore, for accurate evaluation of cytotoxicity based on the surface charge, the material must have high colloidal stability under biological conditions, and the effects of other variables affecting cytotoxicity should be suppressed.

In this study, we designed three differently charged IONPs based on reversible addition–fragmentation chain transfer-mediated (RAFT) polymerization to systematically evaluate the in vitro and in vivo toxicity of charged IONPs. RAFT polymerization has the following advantages: (1) easy introduction of various functional groups based on the same backbone moiety, depending only on the type of monomers used in the polymerization; (2) control of the composition and molecular weight of polymers as desired; and (3) synthesis of polymers with low polydispersity [25]. Three differently charged polymeric ligands—a negative ligand ((−) ligand) using a carboxyl group, a neutral ligand ((n) ligand) using a polyethylene glycol (PEG) moiety, and a positive ligand ((+) ligand) using an amine group—were prepared. The ligands had the same backbone and low polydispersity index (PDI) values. We performed ligand exchange on 12-nm oleic acid-coated IONPs (OAc-IONPs) with the charged polymer ligands, and three charged IONPs were prepared: negatively charged IONPs ((−) IONPs), neutral IONPs ((n) IONPs), and positively charged IONPs ((+) IONPs). The colloidal stability and HD of the charged IONPs were maintained in aqueous conditions and in biological media. The cytotoxicity of the three charged IONPs was evaluated in three different human cell lines—human lung cancer, liver cancer, and neural cancer cell lines—by investigating the changes in viability and the morphological alteration in the cells. In addition, a thorough in vivo toxicity study was performed using serum biochemistry and histological analyses on mice as the target organism. Notably, all of our synthesized IONPs, having superior colloidal stability, showed excellent biocompatibility in vivo and in vitro.

## 2. Experimental Section

### 2.1. Materials

Iron(III) chloride hexahydrate (FeCl_3_·H_2_O, ACS reagent, ≥98%), oleic acid (CH_3_(CH_2_)_7_CH=CH(CH_2_)_7_COOH, technical grade, 90%), 1-octadecene (CH_3_(CH_2_)_15_CH=CH_2_, GC, ≥95%), dimethyl sulfoxide ((CH_3_)_2_SO, HPLC, ≥99.7%), polyethylene glycol methyl ether acrylate (H_2_C=CHCO_2_(CH_2_CH_2_O)nCH_3_, average Mn 480), acrylic acid (CH_2_=CHCOOH, anhydrous, 99%), and thiazolyl blue tetrazolium bromide (MTT) (C_18_H_16_BrN_5_S, Bio reagent, ≥97.5%) were purchased from Sigma Aldrich (St. Louis, MO, USA). Sodium oleate (C_18_H_33_NaO_2,_ >97%), N-[3-(dimethylamino)propyl]acrylamide (C_8_H_16_N_2_O, >98%), and 2-(2-aminoethoxy)ethanol (C_4_H_11_NO_2_, >98%) were purchased from Tokyo Chemical Industry Co., Ltd. (Tokyo, Japan). 2,2′-azobisisobutyronitrile (C_8_H_12_N_4_, GR, 98%) was purchased from Samchun (Seoul, Korea). N-[2-(3,4-Dihydroxyphenyl)ethyl]-2-methylprop-2-enamide (DMA) and dibenzyl trithiocarbonate were synthesized as previously reported [26,27]. A549, Huh-7, and SH-SY5Y were obtained from the American Type Culture Collection (ATCC, Manassas, VA, USA). RPMI 1640, Dulbecco’s modified Eagle’s medium (DMEM), penicillin-streptomycin, and trypsin/EDTA were purchased from Lonza (Walkersville, MD, USA). Fetal bovine serum was purchased from Thermo Fisher Scientific (Rockford, IL, USA). Dulbecco’s phosphate buffered saline (DPBS) was purchased from Welgene (Gyeongsan, Korea).

### 2.2. Synthesis of OAc-IONPs

OAc-IONPs, with high crystallinity and uniform size distribution, were synthesized by a previously reported thermal decomposition method [28]. Briefly, an iron oleate complex (1 mmol) and oleic acid (0.73 mmol) were dissolved in 1-octadecene (1.5 g). The mixture was heated up to 320 °C for 1 h 30 min and maintained at that temperature for 30 min. The nanoparticles were cooled to 20 °C and precipitated using acetone and ethanol mixed solvent (50 mL) by centrifugation, to obtain purified nanoparticles. The sizes and morphologies of the nanoparticles were observed using transmission electron microscopy (TEM).

### 2.3. Synthesis of Differently Charged Polymer Ligands

We synthesized differently charged polymeric ligands through RAFT polymerization, using different functional groups to obtain different charges on the IONPs. For synthesis of the (n) ligand, (−) ligand, and (+) ligand, we used PEG, acrylic acid, and tertiary amine, respectively, as functional groups. All the polymers were equally composed of 20% DMA groups, 60% PEG groups, and 20% functional groups. All monomers (1 mmol) were mixed in N,N-dimethylformamide (1 mL), and dibenzyl trithiocarbonate (RAFT agent, 50 μmol) and 2,2′-azobisisobutyronitrile (50 μmol) were added in a 5-mL ampule. Freeze–pump–thaw cycles were performed three times, and the ampule was sealed under vacuum using a torch gas. The ampule was heated up to 70 °C overnight, and after the reaction, the excess residues were washed with ethyl ether by performing centrifugation thrice. The solvent was removed in a vacuum oven to obtain the polymer products.

### 2.4. Synthesis of Charged IONPs

Ligand exchange was used to modify the OAc-IONPs with the synthesized polymer ligands. We mixed OAc-IONPs (5 mg), each polymer (30 mg), and 2-(2-aminoethoxy)ethanol (100 mg) in CHCl_3_ (500 μL) and allowed the mixture to react for 12 h at 60 °C. After the reaction, the solution was precipitated using diethyl ether by centrifugation (3 min, 1057 g) and dissolved in DIW. To remove the excess reagents, we used a centrifugal filter (MWCO 50 k) and washed the sample thrice.

### 2.5. Characterization

TEM images were observed on a JEM-2100F instrument (JEOL, Tokyo, Japan) operated at 200 kV. The polymers were confirmed by ^1^H nuclear magnetic resonance (NMR) and inverse-gated (IG) ^13^C NMR using an AVANCE III HD (Bruker, Billerica, MA, USA) instrument at 400 MHz using dimethyl sulfoxide-d_6_ solution. The molecular weight and molecular weight distribution were determined using gel permeation chromatography (GPC) (Agilent, Santa Clara, CA, USA). The average HD, distribution, and zeta potential of the IONPs in DIW and cell culture media were determined using a dynamic laser scattering (DLS) particle size analyzer (Nano-ZS90, Malvern Instruments, Malvern, UK). All measurements were recorded in 400-μL disposable cuvettes using a 4-mW He–Ne laser operating at a wavelength of 633 nm at 25 °C, with the scattering angle fixed at 90 °C. Zeta potentials were measured to evaluate the surface charge of the IONPs. Samples were prepared by diluting with DIW or cell culture media as required. The ligands on the surfaces of OAc-IONPs and charged IONPs were confirmed using Fourier transform infrared spectroscopy (FT-IR) on an IRTracer-100 (Shimadzu, Kyoto, Japan).

### 2.6. Cell Lines and Cell Culture

The cell lines used were A549 (human lung cancer cell), Huh-7 (human liver cancer cell), and SH-SY5Y (human neural cancer cell). Each cell was cultured in different suitable media: A549 and Huh-7 were cultured in RPMI 1640 and SH-SY5Y in DMEM supplemented with 10% fetal bovine serum, 100 IU/mL penicillin, and 100 µg/mL streptomycin at 37 °C under 5% CO_2_ in a humidified incubator.

### 2.7. MTT Assay

Cell viability was measured using the MTT assay. The cells were seeded into 96-well flat-bottom plates at starting densities of 1 × 10^4^ cells per well (A549 and Huh-7) and 4 × 10^4^ (SH-SY5Y) cells per well. All cells were cultured for 24 h. The IONPs were treated in each well and incubated for 24 h. Next, the IONPs were washed with DPBS three times, and thiazolyl blue tetrazolium bromide solution (5 mg/mL, 10 μL) was added to each of the 96 wells for 3 h at 37 °C in a CO_2_ incubator. The cells were lysed with dimethyl sulfoxide (150 μL), and absorbance was measured using a microplate reader (GloMax^®^ Discover, Promega, Madison, WI, USA) at 560 nm.

### 2.8. Animals

The experimental design using animals were reviewed and approved by the Association for the Assessment and Accreditation of Laboratory Animal Care (AAALAC) International and the Institutional Animal Care and Use Committee (IACUC) of the Korea Institute of Toxicology (approval no. 1605–0174). Balb/c mice (male, 6 weeks) were purchased from Orient-Bio Co. (Seongnam, Korea) and were adapted for seven days under environmentally controlled animal room conditions of 23 ± 3 °C, 50 ± 20% relative humidity, and 10–20 times/h air ventilation with a 12/12-h light/dark cycle. Standard food pellets and water were provided ad libitum. The mice were randomly grouped and were intravenously injected with the IONPs at doses of 2 mg Fe/kg (low dose, *n* = 3) and 10 mg Fe/kg (high dose, *n* = 3), to observe acute toxicity. 1× PBS was used as the control vehicle. All mice were sacrificed after one day of treatment, and then their body weights and target organ weights (liver, kidney, lung, heart, spleen, and brain) were calculated.

### 2.9. Hematological Analysis and Histological Analysis

Blood was collected from the caudal vena cava, and serum was obtained by centrifugation at 1057g for 10 min at room temperature. Blood urea nitrogen (BUN), creatinine (CREA), alanine aminotransaminase (ALT), alkaline phosphatase (ALP), aspartate aminotransaminase (AST), and total bilirubin (TBIL) were measured using an automatic TBA120FR NEO analyzer (Toshiba Co., Tokyo, Japan). The mean values were compared, and the statistical significance was calculated using Student’s *t*-test. For histopathological analyses, the target organs were isolated, fixed in 10% neutral-buffered formalin, and embedded in paraffin. The specimens were cut into 4-µm-thick slices using a microtome (RM2165, Leica, Germany). The sections were stained with hematoxylin and eosin and examined under a light microscope (Nikon E-400, Tokyo, Japan). All histopathological results were blindly reviewed by pathology experts.

### 2.10. Statistical Analysis

All data are presented as the means ± SD for cell viability of IONPs. Statistically significant differences between groups were analyzed using one-way ANOVA with GraphPad Prism software (GraphPad Inc., San Diego, California, USA city, state abbreviation, USA). *p* < 0.05 indicated a statistically significant difference between groups.

## 3. Results and Discussion

### 3.1. Synthesis and Characterization of Differently Charged Ligands

**Figure 1:**
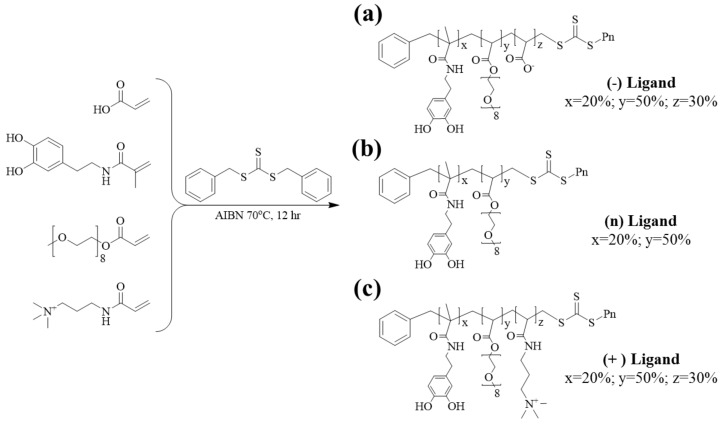
RAFT polymerization reaction for synthesis of different charged polymer ligands. (**a**) (−) ligand, (**b**) (n) ligand, and (**c**) (+) ligand.

We designed and synthesized differently charged ligands via RAFT polymerization. The ligands exhibited multidentate anchoring groups and hydrophilic groups, which improved the colloidal stability and hydrophilic properties of the IONPs. The synthesized ligands were composed of three groups: an anchoring group, functional groups, and hydrophilic groups. Catechol-derived DMA was used as the anchoring group and had a strong binding affinity for the Fe ions on the IONPs. The functional group provided different charges on the IONPs. The carboxyl group (acrylic acid) was used as a negative charge, and a tertiary amine group ((N-[3-(dimethylamino)propyl]acrylamide)) was used as the positive charge. A substance containing PEG was used as the hydrophilic group because the hydrophilic properties of PEG improve the dispersibility of the IONPs in aqueous solutions. In general, PEG provides several advantages, such as increased biocompatibility, reduced immunogenicity, and stability between particles via steric repulsion [29]. It also reduces the adsorption of various plasma proteins [30] and enables circulation in the body for a long duration in vivo. Therefore, PEG is an ideal coating material for bio-applications [31,32]. We obtained the final polymer with 20% anchoring groups (DMA), 50% hydrophilic groups (PEG moieties), and 30% charged functional groups or PEG moiety for the charged part of IONPs, respectively (Figure 1). This specific polymer design was used because the dispersibility of the nanoparticles in cell culture media was remarkably reduced. In the case of the ligands composed of 20% anchoring group and 80% positive functional group without PEG, significant aggregation was observed, although the nanoparticles were well-dispersed in the aqueous solution (Figure S1).

**Figure 2:**
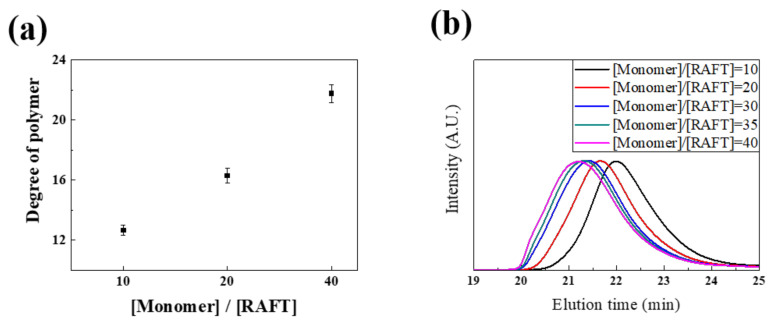
RAFT polymerization of (n) ligand showing (**a**,**b**) controllable polymer DP as a [Monomer] to [RAFT] ratio. GPC of (n) ligand in THF, showing a low PDI with a [Monomer]:[RAFT] ratio of 20:1 and [AIBN]:[RAFT] ratio of 1:1 (red line), and poor PDI without RAFT agent (black line).

Prior to the synthesis of charge-modulated ligands, we checked whether the RAFT polymerization system could successfully synthesize the (n) ligand. The molecular weight of the polymer was well controlled by using the RAFT agent. When the RAFT agent was used, the PDI value was 1.26, and it was 1.59 without the RAFT agent (Figure S2). In addition, the degree of polymerization (DP) of the (n) ligand increased as the [Monomer]:[RAFT] ratio increased, although there was a slight difference between the theoretical [Monomer]:[RAFT] ratio and the measured DP of the (n) ligand analyzed by GPC (Figure 2a,b). We speculated that this result was caused by the effect of catechol on radical scavenging characteristics. Previous studies on the DOPA moiety including a polymer have reported that propagating radicals during polymerization can be decreased by catechol, consequently limiting the polymer molecular weight [33,34]. In addition, we confirmed that the composition of each monomer can be controlled via a proton NMR. The monomer ratio in the polymer could be modulated by RAFT polymerization. In Table 1, we show that the [DMA]:[PEG] ratio in the polymer was well controlled, as desired. We synthesized the (n) ligand with various DMA proportions of 10%, 20%, 30%, 40%, and 50%. After synthesizing the (n) ligand, the calculated proportion of DMA was observed to be 9%, 20%, 28%, 35%, and 45%, respectively, by ^1^H-NMR analysis. The calculated ratio of DMA to PEG in the synthesized polymer nearly corresponded with the theoretical ratio. These data agree well with previously reported studies about RAFT mediated synthetic DOPA based polymers [35]. Based on these results, three charge-modulated ligands were synthesized, and it was confirmed that each monomer was polymerized at the desired ratio by the ^1^H-NMR spectra, as shown in Figure S3. Distinct proton peaks of DMA, PEG, and tertiary amine were observed at 6.3–6.7 ppm, 3.44–3.54 ppm, and 2.07–2.22 ppm by ^1^H-NMR spectroscopy, respectively. The synthesized random copolymers with the three controlled charges and their molecular characteristics are summarized in Table 2. In case of the (-) ligand, we could not accurately calculate the ratio of the functional group because no distinctive proton peaks of acrylic acid were observed by ^1^H-NMR. Therefore, its ratio was confirmed by IG ^13^C-NMR (Figure S4). In addition, it was verified indirectly after the EDC coupling of the acrylic acid of the (n) ligand with N-hydroxysuccinimide (NHS) (Figure S5). Proton peaks of NHS were observed at 2.59 ppm by ^1^H-NMR after the conjugation of NHS on the (−) ligand with different ratios of acrylic acid (10%, 20%, and 30%). The molecular weight of the (+) ligand measured by the GPC was lower than that calculated by ^1^H-NMR [28].

### 3.2. Synthesis of Charged IONPs Dispersed in the Aqueous Solution

**Figure 3:**
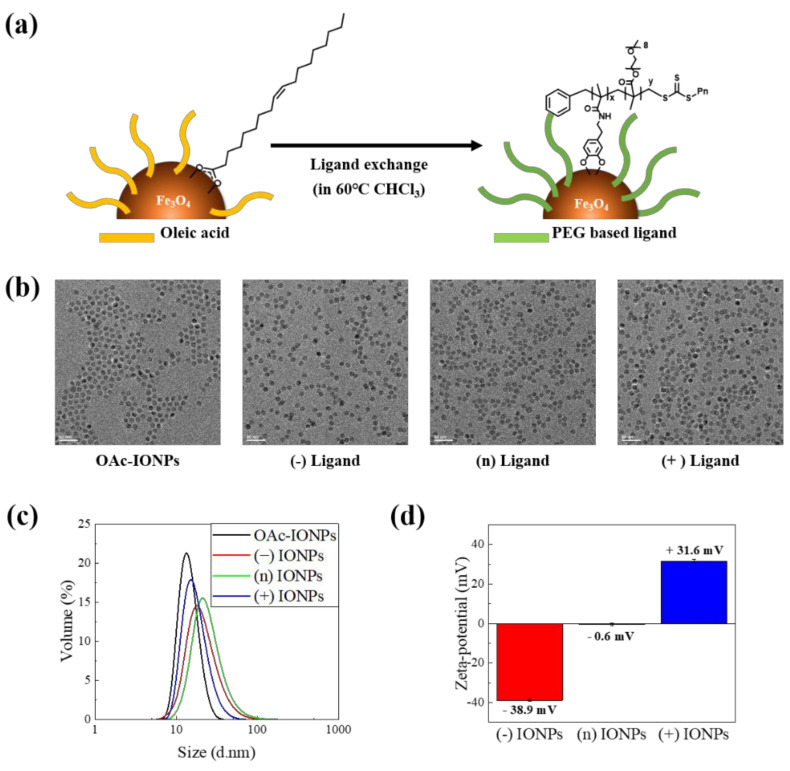
Characterization of IONPs after ligand exchange. (**a**) Schematic of ligand exchange of OAc-IONPs with charged ligands, (**b**) TEM images of OAc-IONPs dispersed in hexane and three charged IONPs dispersed in DIW. (**c**) HD of OAc-IONPs and the three charged IONPs, and (**d**) zeta-potential of the three charge IONPs.

To investigate the effects of charges of IONPs, we synthesized monodisperse OAc-IONPs using a thermal decomposition method that can control the size, polydispersity, and shape of the NPs with large quantities to exclude the effect of size [23]. Next, the surface modification step of the initial OAc-IONPs with hydrophilic ligands is essential for their application in a biomedical environment because OAc-IONPs can only be dispersed in organic solvents such as hexane and chloroform. Therefore, we used a ligand exchange method to convert the hydrophobic material initially synthesized in the organic solvent into a water-dispersible hydrophilic material, as shown in Figure 3a. Ligand exchange has the advantage of simply compacting ligands without significantly increasing HD [36]. It is very important to coat the surface uniformly and compactly for the accurate analysis of surface characteristics and reproducible synthesis of IONPs. We could also consistently evaluate the toxicity under the proposed ligand exchange method. As shown in Figure 3b, the TEM results showed that the OAc-IONPs (about 11.6 ± 0.7 nm) and three surface-modified IONPs were dispersed uniformly. The HD of OAc-IONPs was characterized by the DLS measurements, which confirmed that the size of the OAc-IONPs was approximately 14.58 nm and the PDI value was 0.046. In addition, the synthesized compact and uniform IONPs showed no change in HD between OAc-IONPs and the three charged IONPs (Figure 3c). The PDI values were low (0.25–0.3). The surface charge of each charged IONP was measured using the zeta-potential, as shown in Figure 3d. The surface charges of (−) IONPs, (n) IONPs, and (+) IONPs were −39 mV, −0.6 mV, and +32 mV, respectively. Figure S6 shows the characterization of the surface for OAc-IONPs and three charged IONPs by FT-IR spectroscopy. After ligand exchange with polymer ligands, an aryl band and phenol alcohol band in catechol were observed at 1446 cm^−1^ and 1267 cm^−1^, respectively, and C-O-C stretching in PEG was observed at 1101 cm^−1^ [37]. In addition, the disappearance of stretching modes of –CH_2_- and –CH_3_ in oleic acid (2850 and 2920 cm^−1^) in OAc-IONPs was obvious evidence of ligand exchange [38]. For (+) IONPs, a sharp peak was observed at 1671 cm^−1^, compared with that of (−) and (n) IONPs because of the high contents of amide in the (+) ligand, which is synthesized from N-[3-(dimethylamino)propyl]acrylamide, and a slight C–N stretching was observed in the tertiary amine at 1200 cm^−1^.

### 3.3. Stability of IONP in DIW and Cell Culture Media

**Figure 4:**
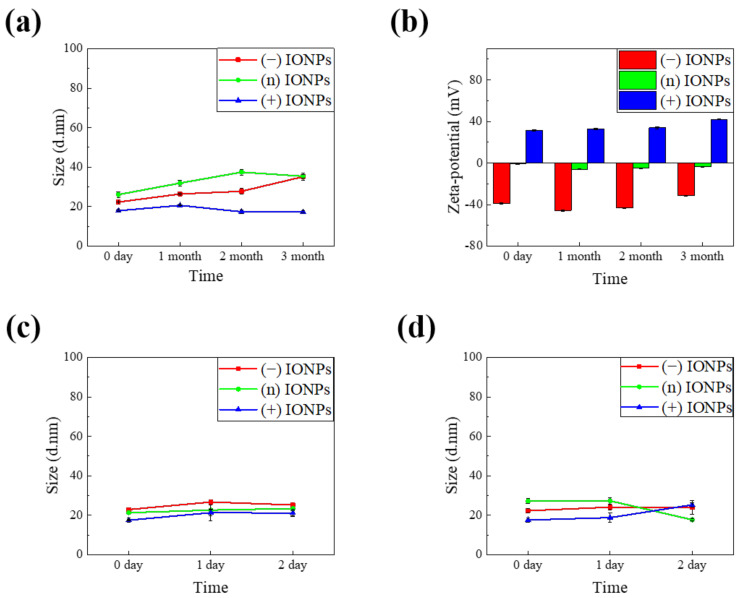
Colloidal stability of charged IONPs in DIW for three months and in cell culture media for two days. (**a**) HD and (**b**) zeta-potential of three differently charged IONPs in DIW for three months. (**c**) HD of three differently charged IONPs in RPMI 1640 medium and (**d**) in DMEM for two days.

It is important to maintain the stable properties of nanoparticles under aqueous conditions. Although the synthesized nanoparticles exhibit initial colloidal stability, they may become unstable because of changes in physical properties depending on the storage period and storage method, which affect the toxicity of IONPs. First, we confirmed long-term stability of the IONPs in DIW. The IONPs were refrigerated at 4 °C sealed with a parafilm and wrapped in foil to stored it for a long time to minimize changing the physical properties of the samples. We tested the stability of the IONPs dispersed in DIW for three months by DLS and zeta-potential measurement. In Figure 4a, the HD of the (−) IONPs and the (n) IONPs shows a very subtle increase in DIW. The changes in HD of the (−) IONPs and (n) IONPs were ±5.36 nm and ±4.98 nm over the three months, respectively. Moreover, the (+) IONP remained constant at ±1.57 nm with almost no change in size. In Figure 4b, the zeta-potential values of (−) IONPs, (n) IONPs, and (+) IONPs are ±6.24 mV, ±2.31 mV, and ±4.77 mV, respectively. Notably, all the synthesized IONPs with polymer ligands showed highly stable size and surface charge.

We further studied the stability of IONPs in the cell culture medium to understand the effect of many factors on the physicochemical properties of nanomaterials, such as proteins, salts, and various pH conditions. To test the stability of IONPs in the cell culture conditions, we evaluated the stability test of the IONPs under two cell culture media of the RPMI 1640 medium and DMEM and 10% FBS was added into each medium. The samples were incubated at 37 °C for two days and analyzed the HD by DLS, as shown in Figure 4c,d, respectively. In general, charged substances tend to aggregate or form protein corona in a medium [19,39]. Surprisingly, however, all of our charged IONPs showed no change in their size for two days in both the RPMI 1640 medium and DMEM. We also observed the excellent long-term stability of the three IONPs in cell culture media during three months (Figure S7). This indicates that all the differently charged IONPs were sufficiently stable in order to conduct in vitro tests. The high stability of IONPs is attributed to the robust binding affinity between catechol and iron ions owing to the presence of multi-anchor groups. In addition, introduction of a PEG moiety and adequate charged groups to the surface of IONPs optimizes nanoparticles stability via the combination of steric hindrance and charge–charge repulsion. Therefore, the toxicity of the three types of charged IONPs could be accurately evaluated by minimizing the variable factors derived from material properties, which is addressed in the next section.

### 3.4. Evaluation of In Vitro Toxicity of Differently Charged IONPs

**Figure 5:**
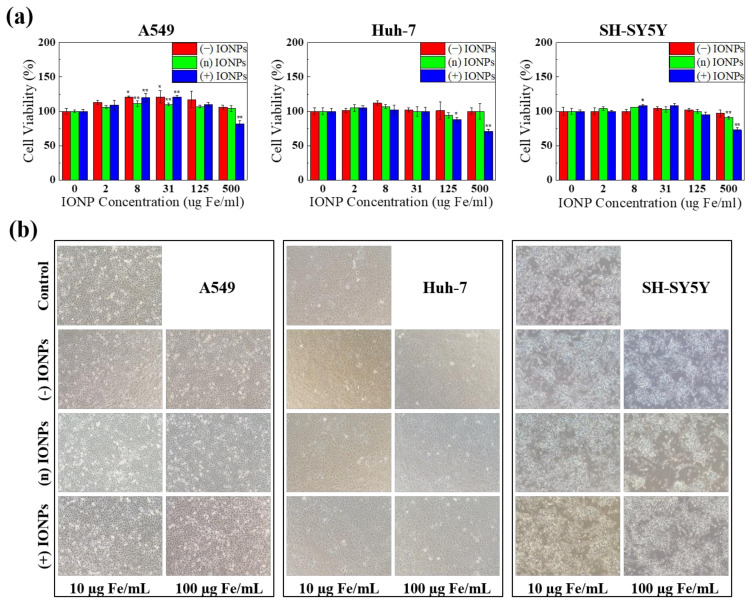
(**a**) Effects of the three charged IONPs on the viabilities of three different cell lines, A549, Huh-7, and SH-SY5Y. All cells were exposed for 24 h to increasing concentrations upon 500 µg Fe/mL. Cell viability was analyzed by MTT assay. * *p* ≤ 0.05 and ** *p* ≤ 0.01 compared to the controls (Dunnett test with a one-way ANOVA). (**b**) Changes in cell morphology after treatment with 10 and 100 μg Fe/mL of the three charged IONPs by microscopy.

To determine if the surface charge of IONPs influences the cytotoxicity of IONPs, we conducted an MTT assay using three human cell lines—A549, Huh-7, and SH-SY5Y—in three target organs: lung, liver, and nerve [40]. After each cell was treated with various concentrations of differently charged IONPs (0, 2, 8, 31, 125, and 500 µg Fe/mL) for 24 h, the adsorption of formazan was measured at 560 nm (Figure 5a). In each cell line, all IONPs showed similar tendencies of cell viability. The viability slightly decreased with increasing concentrations of IONPs. Although it is generally known that positively charged nanoparticles are absorbed at a high rate because of the electronic interaction with the cell membrane, which consequently leads to serious toxicity. However, in our study, (+) IONPs reduced cell viability by approximately 30% only at high concentrations. Therefore, we chose the maximum concentration as 500 µg Fe/mL with 100-fold bio-applicable concentration (Cmax. 5.5 ± 0.6 μg/mL) from the commercially available IONPs (Feridex IV, Bayer Healthcare Pharmaceuticals, Leverkusen, Germany).

Furthermore, morphological observation evidenced that there were no significant alterations in A549, Huh-7, and SH-SY5Y at 10 µg Fe/mL and 100 µg Fe/mL of treatment for 24 h (Figure 5b). These results indicate that the differently charged IONPs are rarely toxic below excess concentration and there was no significant discrepancy in the effects of charged IONPs on cytotoxicity.

### 3.5. Evaluation of In Vivo Toxicity of Differently Charged IONPs

**Figure 6:**
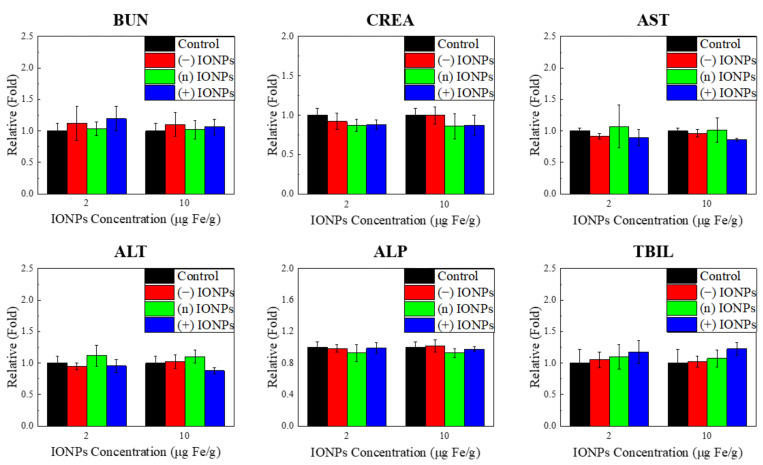
Blood chemistry of mice following injection of three charged IONPs at 2 µg/g and 10 µg/g for 24 h. (*n* = 3) Six parameters were analyzed. BUN and CREA were related to the kidney function, and AST, ALT, ALP, and TBIL were related to the liver function.

To observe the in vivo toxicity of three differently charged IONPs in mice, we conducted hematological analysis and histopathological observation. For the in vivo toxicity testing, the dosage level of over 10-fold bio-applicable concentration and the same administration route were determined on the basis of the commercially available IONPs. For the magnetic imaging in the clinical application, 0.56 mg Fe/kg of IONPs was singly administered via intravenous injection for several hours (Feridex IV). Here, the three differently charged IONPs were administered in mice at 2 mg Fe/kg and 10 mg Fe/kg via intravenous injection. Serum biochemistry indicated that there were no changes in the toxicity markers for all IONPs; BUN and CREA for the kidney and ALT, ALP, AST, and TBIL for the liver (Figure 6). Histopathological observation indicated that no significant lesions were present on the liver, kidney, lung, heart, spleen, and brain (Table 3). For 10 Fe mg/kg of (n) IONPs, few inflammatory cell foci were observed in the liver; however, a significant injury was not satisfactorily confirmed on the basis of the historical data of controls (Table 3, Figure S8). These results indicate that charged IONPs can be applied as biocompatible materials to various biomedical fields by controlling the surface ligands and adjusting the usage amount depending on the purposes.

## 4. Conclusions

We have successfully designed charged IONPs with high colloidal stability and systematically evaluated their cytotoxicity in three human cell lines—A549, Huh-7, and SH-SY5Y. Through RAFT polymerization and ligand exchange, we prepared three differently charged IONPs with DMA as the multiple anchoring group and various functional ligands—hydrophilic PEG polymer, negative carboxylic acid, and positive quaternary amine. All the charged IONPs were monodispersed with narrow size distribution in aqueous solution and exhibited high colloidal stability for up to three months in DIW and two days in the RPMI 1640 medium and DMEM without agglomeration. Depending on the concentration of IONPs, toxicity was assessed in vitro in three cell lines (lung, liver, and brain) via cell viability assays and morphological observations. In MTT assay, all the charged IONPs showed no significant toxicity in each cell line up to the maximum concentration of 500 µg Fe/mL and cell morphologies showed no changes when treated with 100 µg Fe/mL IONPs. For in vivo tests, we performed hematological and histological analyses in mice; the results showed that the three IONPs showed no significant toxicity and were biocompatible. These results suggested that the charged IONPs with high stability and less toxicity under physiological conditions have potential for application in various biomedical fields.

## Figures and Tables

**Table 1:** Theoretical and calculated values of [DMA]:[PEG] ratio in the (n) ligand analyzed by ^1^H-NMR measured in CDCl_3_.

Theoretical Value		Calculated Value	
DMA	PEG	DMA	PEG
10	90	9	91
20	80	20	80
30	70	28	72
40	60	35	65
50	50	45	55

**Table 2:** Summary of characterization of the three charged ligands.

Sample	DMA:PEG:Functional Group		Mn		DP ([Monomer]/[RAFT])		
	Theoretical	Experimental (NMR)	NMR	GPC (PDI)	Theoretical	NMR	GPC
(−) ligand	20:50:30	17:52:31	4738	5253 (1.28)	20	12	13
(n) ligand	20:80:0	18:82:0	9745	7049 (1.29)	20	22	16
(+) ligand	20:50:30	22:53:25	7239	2509 (1.25)	20	20	7

**Table 3:** Histological observation of six main organs in mice for three differently charged IONPs.

	PBS	(−) IONPs		(n) IONPs		(+) IONPs	
		2 Fe mg/kg	10 Fe mg/kg	2 Fe mg/kg	10 Fe mg/kg	2 Fe mg/kg	10 Fe mg/kg
Liver Inflammatory cell foci				+(3)	+(1), ++(2)		
Kidney			+(1)				
Basophilic tubules		+(1)		+(1)			
Inflammatory cell foci		+(1)		+(1)			
Mineralization					+(1)		
Lung							
Heart Cardiomyopathy						+(1)	
Spleen							
Brains							

Tissues with diagnoses (*n* = 3). +, minimum or very slight degree present. ++, slight degree or small amount present.

## Supplementary Materials

The following are available online at https://www.mdpi.com/article/10.3390/nano11113068/s1, Figure S1: Stability test of IONPs coated with ligands having 80% positively functional groups in DIW and RPMI 1640 media. (a) H.D. of the IONPs dispersed in water and RPMI 1640 medium. Camera image of the IONPs (b) well-dispersed in water and (c) agglomerated in RPMI 1640 medium, Figure S2: GPC of (n) ligand in THF showing narrow PDI with a [Monomer]: [RAFT] ratio of 20:1 and [AIBN]: [RAFT] ratio of 1:1 (black line), along with poor PDI without a RAFT agent (red line), Figure S3: ^1^H-NMR spectra of the three polymeric ligands measured in DMSO-d6, Figure S4: IG ^13^C NMR spectra of (−) ligand and (n) ligand measured in DMSO-d6. The composition of DMA + PEG and functional group in the (−) ligand were 48% and 52%, respectively, by IG ^13^C NMR and those of DMA and PEG in the (−) ligand were 25% and 75%, respectively, by ^1^H NMR. Based on these data, we finally calculated the proportion of DMA, PEG, and the functional group of (n) ligand to be 17%, 52%, and 31%, respectively. The compositions of DMA and PEG in the (n) ligand were 18% and 82%, respectively, by IG ^13^C NMR and it corresponded with the ^1^H NMR data. These results support the validity of the (−) ligand data, Figure S5: ^1^H-NMR spectra of (−) ligand (a) with different ratios of acrylic acid and (b) after conjugation with NHS measured in DMSO-d6, Figure S6: FT-IR spectra of (a) the three charged OAc-IONPs and (b) three charged IONPs after the ligand exchange of OAc-IONPs, Figure S7: Colloidal stability of three charged IONPs in cell culture media. H.D of three charged IONPs (a) in RPMI1640 media and (b) in DMEM media until 3 month, Figure S8: Histological image of liver and kidney in mice for three differently charged IONPs. Magnification = X200, Scale bar = 100 μm.

## Abbreviations

IONPs	iron oxide nanoparticles
RAFT polymerization	reversible addition–fragmentation chain transfer-mediated polymerization
HD	hydrodynamic diameter
(−) ligand	negative ligand
(n) ligand	neutral ligand
PEG	polyethylene glycol
(+) ligand	positive ligand
PDI	polydispersity index
OAc-IONPs	oleic acid-coated IONPs
(−) IONPs	negatively charged IONPs
(n) IONPs	neutral IONPs
(+) IONPs	positively charged IONPs
MTT	thiazolyl blue tetrazolium bromide
DMA	4-Dihydroxyphenyl)ethyl]-2-methylprop-2-enamide
DMEM	Dulbecco’s modified Eagle’s medium
DPBS	Dulbecco’s phosphate buffered saline
TEM	transmission electron microscopy
NMR	nuclear magnetic resonance
GPC	gel permeation chromatography
DLS	dynamic laser scattering
FT-IR	Fourier transform infrared spectroscopy
BUN	Blood urea nitrogen
CREA	creatinine
ALT	alanine aminotransaminase
ALP	alkaline phosphatase
AST	aspartate aminotransaminase
TBIL	total bilirubin
DP	degree of polymerization
NHS	N-hydroxysuccinimide
