# Supplementary material for: Charge-Modulated Synthesis of Highly Stable Iron Oxide Nanoparticles for In Vitro and In Vivo Toxicity Evaluation
Source: Nanomaterials (Basel). 2021 Nov 14;11(11):3068. doi: 10.3390/nano11113068 (PMC8624538; doi:10.3390/nano11113068)
Supplement: Supplementary file 1 [file nanomaterials-11-03068-s001.zip › nanomaterials-1435925 supplementary.pdf]

nanomaterials

MDPI

Supplementary Materials

Charge-Modulated Synthesis of Highly Stable Iron Oxide Nanoparticles for In Vitro and In Vivo Toxicity Evaluation

Sunyoung Woo [1], Soojin Kim [2,3], Hyunhong Kim [1], Young Woo Cheon [4], Seokjoo Yoon [2,5,*], Jung-Hwa Oh [2,5,*] and Jongnam Park [1,6,*]

[1] School of Energy and Chemical Engineering, Ulsan National Institute of Science and Technology (UNIST), Ulsan 44919, Korea; sywoo1225@unist.ac.kr (S.W.); khh2008@unist.ac.kr (H.K.)
[2] Department of Predictive Toxicology, Korea Institute of Toxicology (KIT), Daejeon 34114, Korea; sjkim@kitox.re.kr
[3] Department of Bio and Brain Engineering, Korea Advanced Institute of Science and Technology (KAIST), Daejeon 34141, Korea
[4] Department of Plastic and Reconstructive Surgery, Gachon University Gil Medical Center, Incheon 21565, Korea; youngwooc@gilhospital.com
[5] Department of Human and Environmental Toxicology, University of Science and Technology (UST), Daejeon 34113, Korea
[6] Departmento of Biomedical Engineering, Ulsan National Institute of Science and Technology (UNIST), Ulsan 44919, Korea
* Correspondence: sjyoon@kitox.re.kr (S.Y.); jhoh@kitox.re.kr (J.-H.O.); jnpark@unist.ac.kr (J.P.)

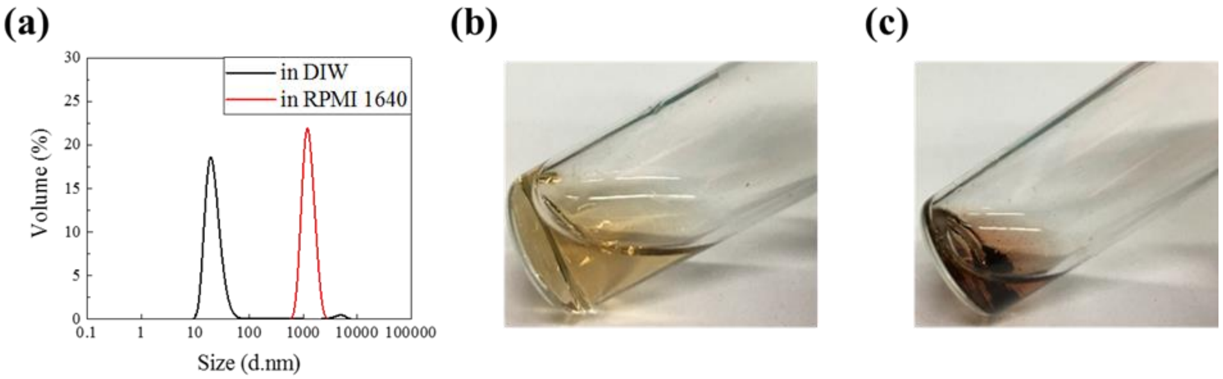

Figure S1. Stability test of IONPs coated with ligands having 80% positively functional groups in DIW and RPMI 1640 media. (**a**) H.D. of the IONPs dispersed in water and RPMI 1640 medium. Camera image of the IONPs (**b**) well-dispersed in water and (**c**) agglomerated in RPMI 1640 medium.

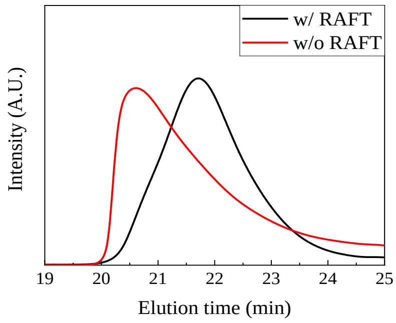

Figure S2. GPC of (n) ligand in THF showing narrow PDI with a [Monomer]: [RAFT] ratio of 20:1 and [AIBN]: [RAFT] ratio of 1:1 (black line), along with poor PDI without a RAFT agent (red line).

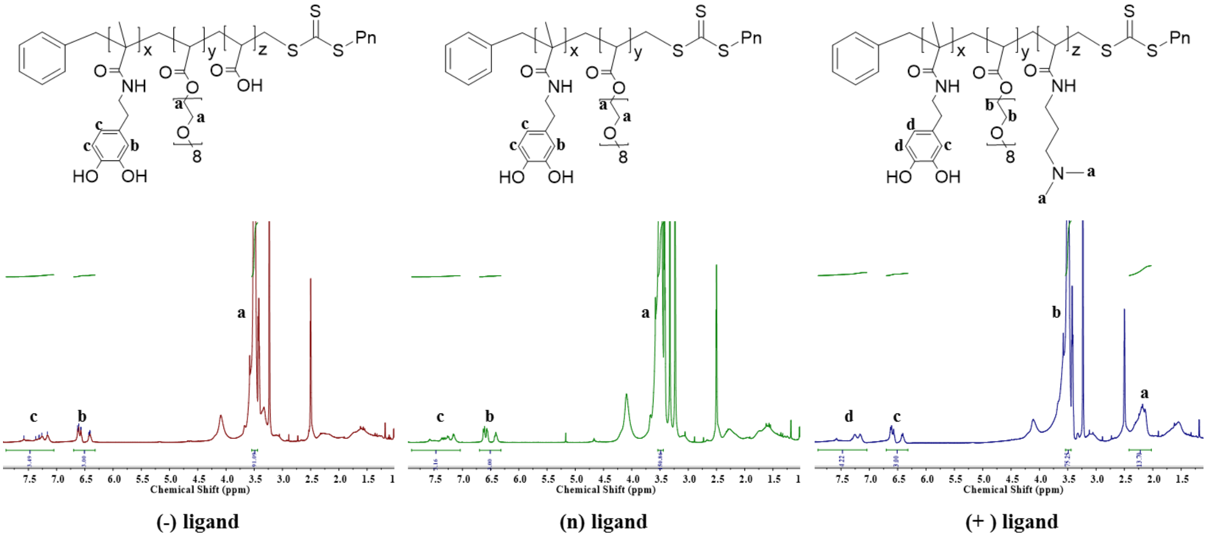

Figure S3. ^{1}H-NMR spectra of the three polymeric ligands measured in DMSO-d6.

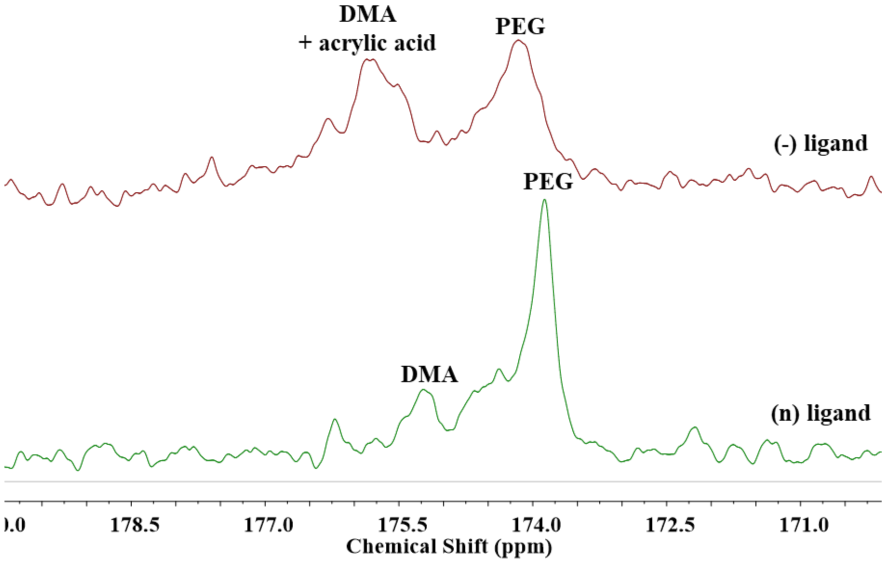

Figure S4. IG ^{13}C NMR spectra of (−) ligand and (n) ligand measured in DMSO-d6. The composition of DMA + PEG and functional group in the (−) ligand were 48% and 52%, respectively, by IG ^{13}C NMR and those of DMA and PEG in the (−) ligand were 25% and 75%, respectively, by ^{1}H NMR. Based on these data, we finally calculated the proportion of DMA, PEG, and the functional group of (n) ligand to be 17%, 52%, and 31%, respectively. The compositions of DMA and PEG in the (n) ligand were 18% and 82%, respectively, by IG ^{13}C NMR and it corresponded with the ^{1}H NMR data. These results support the validity of the (−) ligand data.

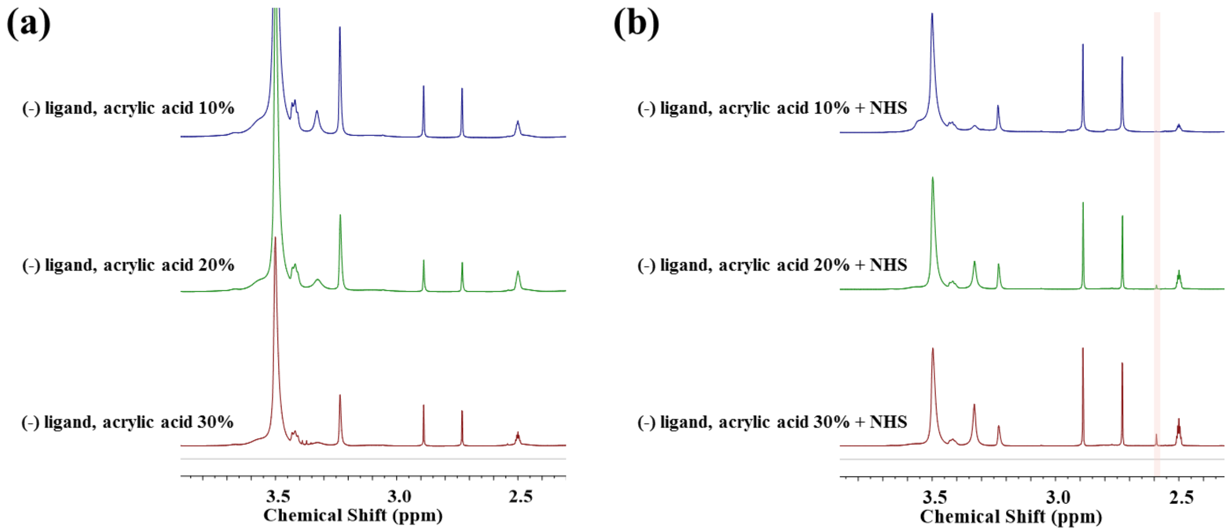

Figure S5. ^{1}H-NMR spectra of (−) ligand (**a**) with different ratios of acrylic acid and (**b**) after conjugation with NHS measured in DMSO-d6.

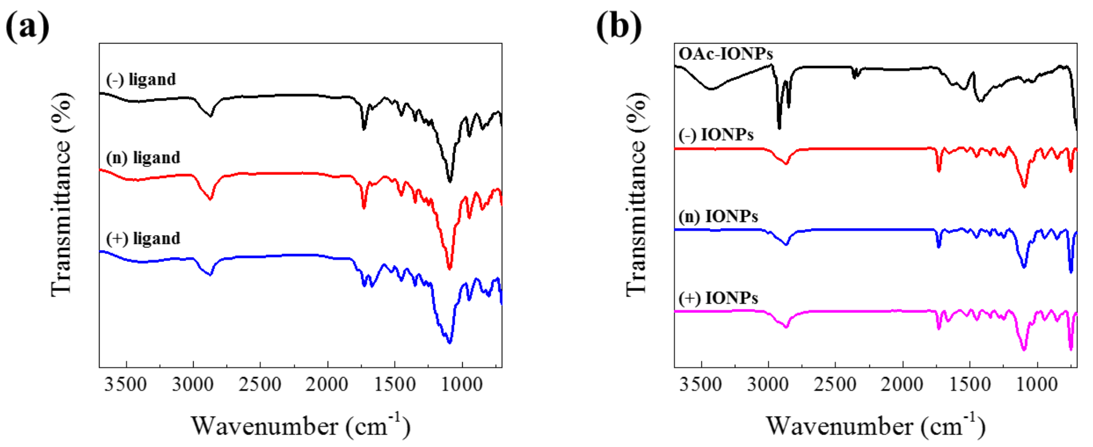

Figure S6. FT-IR spectra of (**a**) the three charged OAc-IONPs and (**b**) three charged IONPs after the ligand exchange of OAc-IONPs.

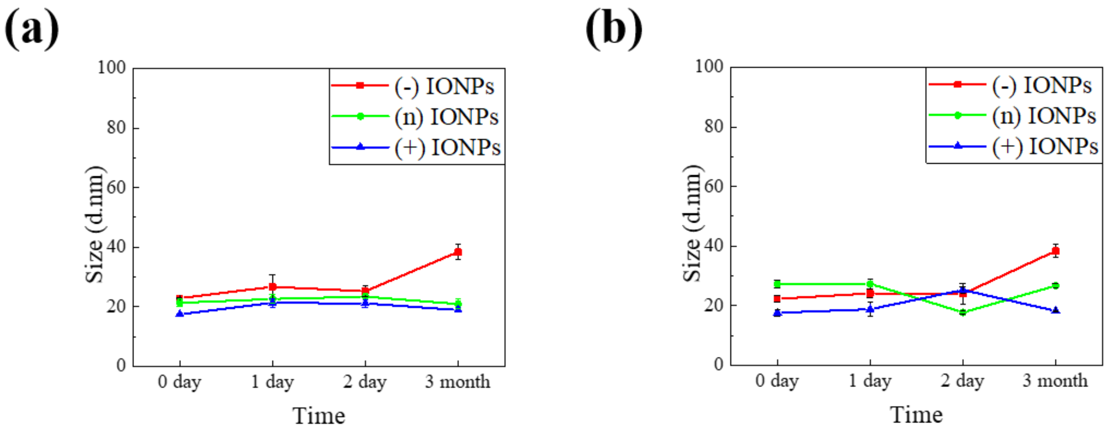

Figure S7. Colloidal stability of three charged IONPs in cell culture media. H.D of three charged IONPs (**a**) in RPMI1640 media and (**b**) in DMEM media until 3 month.

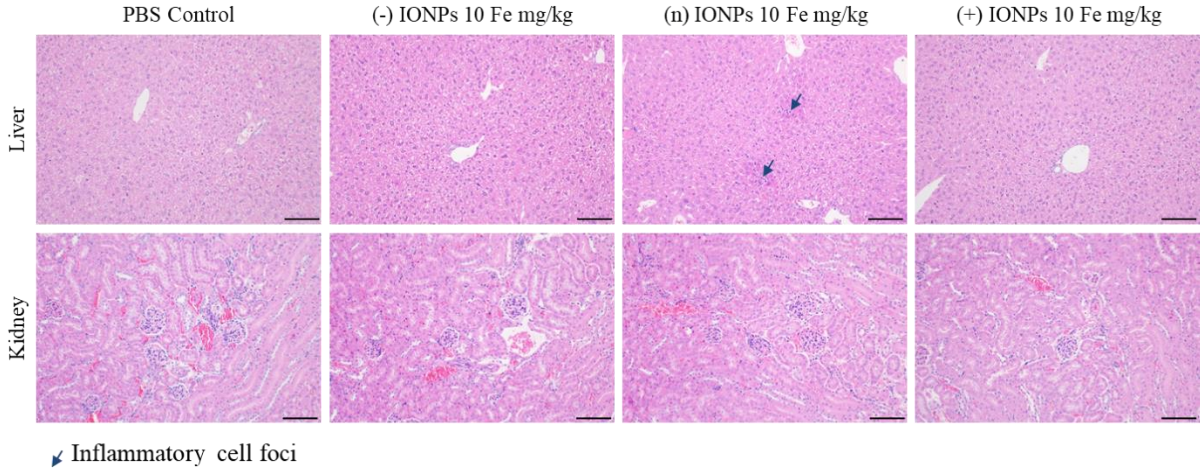

Figure S8. Histological image of liver and kidney in mice for three differently charged IONPs. Magnification = X200, Scale bar = 100 μm.
